# Facing COVID-19 in Georgia - left behind children coping better with COVID-19 precautions

**DOI:** 10.1093/eurpub/ckac131.066

**Published:** 2022-10-25

**Authors:** K Antia, A Berner-Rodoreda, V Winkler

**Affiliations:** Heidelberg Institute of Global Health, Heidelberg University Hospital, Heidelberg, Germany

## Abstract

**Background:**

Georgia, a major migrant sending country, with about 39% of children living with their caregivers while at least one of the parents migrated (left-behind children, LBC) has been severely affected by the COVID-19 pandemic. The main aim of this study was to qualitatively explore LBC’s perception and experience during the pandemic.

**Methods:**

Between December 2021 and January 2022, we conducted 39 (29 LBC, 10 non-LBC) individual in-depth interviews with schoolchildren aged 12-18 in a public school from a migrant sending region. We conducted life history narrative interviews and used a thematic analysis approach.

**Results:**

Preliminary findings show four salient themes: (1) Family members’ first reactions to the pandemic influence children’s emotional health irrespective of parents’ working arrangements. Children express less stress and anxiety when families show emotional stability and are not overwhelmed by the pandemic; (2) All interviewed children find COVID-19 and home-schooling a challenge. (3) LBC express more intense fear about infecting their grandparents than non-LBC. (4) Closer family ties to parents and siblings and access to better equipment help LBC to cope better than non-LBC. LBC view a positive side of Covid-19 in being able to enjoy more time with a parent, who would have otherwise worked abroad.

**Conclusions:**

Overall, all children perceive the COVID-19 pandemic as a challenge, yet closeness with a returned parent and with siblings and more affluence helps LBC to cope better than non-LBC with COVID-19 precautionary measures like home-schooling.

**Key messages:**

• All children are affected by the COVID-19 pandemic. Emotional stability in the family is important in fostering resilience and coping mechanisms among children.

• LBC experience the added benefit of better equipped homes and enjoy the presence of the migrant parent at home.

